# Location and Visualization of Working p-n and/or n-p Junctions by XPS

**DOI:** 10.1038/srep32482

**Published:** 2016-09-01

**Authors:** Mehmet Copuroglu, Deniz Caliskan, Hikmet Sezen, Ekmel Ozbay, Sefik Suzer

**Affiliations:** 1 Department of Chemistry, Bilkent University, 06800 Ankara, Turkey; 2 Nanotechnology Research Center, Department of Electrical and Electronics Engineering and Department of Physics, Bilkent University, 06800, Ankara, Turkey

## Abstract

X-ray photoelectron spectroscopy (XPS) is used to follow some of the electrical properties of a segmented silicon photodetector, fabricated in a p-n-p configuration, during operation under various biasing configurations. Mapping of the binding energy position of Si2p reveals the shift in the position of the junctions with respect to the polarity of the DC bias applied. Use of squared and triangular shaped wave excitations, while recording XPS data, allows tapping different electrical properties of the device under normal operational conditions, as well as after exposing parts of it to harsh physical and chemical treatments. Unique and chemically specific electrical information can be gained with this noninvasive approach which can be useful especially for localized device characterization and failure analyses.

The p-n junction is the backbone of electronic and optoelectronic devices^1^. Therefore, the capability to follow its fabrication, detection, location, identification, as well as characterization under realistic operational conditions are all relevant issues that have captured immense attention over more than one century^2^. In the past, mostly electrical current-based techniques have been used for the identification and/or characterization of devices, which usually include a number of different electronic building blocks, such as diodes, transistors, etc. In addition, advanced and powerful analytical techniques are increasingly being used/developed for probing the chemical and physical as well as electrical properties of materials and devices under realistic operating conditions, such as electron based microscopies (e.g. scanning electron microscopy (SEM)), or other imaging techniques with submicron lateral resolution. The first demonstration was reported by Chang and Nixon, revealing a measurable change in the secondary electron yield across a p-n junction^3^. Later, Kaetsner *et al*.^4^ used SEM to report on a direct observation of the potential variations inside a 90 nm GaAs channel^5^, and Li *et al*. reported a similar observation in carbon nanotubes^6^. Recently, scanning transmission electron microscopy (STEM) using differential phase-contrast has also been proven to reveal the built-in electric field at a p-n junction^7^. Recent SEM characterizations of GaN nanorods have extended conventional mapping by adding *in-situ* electrical biasing thus enabling the direct imaging of the p-n junction under operational conditions and yielding critical information on its depletion width^8^,^9^. Other techniques like cathode-luminescence in a scanning transmission microscope (CL-STEM)^10^ and conductive atomic force microscopy (AFM) have also been utilized for the characterization of the p-n junction in GaN nanorods^11^ and porous GaN based LEDs^12^. The properties of p-n junctions in photodiodes and photovoltaics have also been extensively investigated using a combination of electrical and optical analyses tools^13^,^14^,^15^,^16^,^17^,^18^,^19^.

All of these powerful techniques provide limited chemical information, if any. On the other hand, electron spectroscopic techniques, such as Auger electron spectroscopy (AES) and x-ray photoelectron spectroscopy (XPS), in addition to their chemical sensitivity, have the ability to reflect the electrical potential of the medium surrounding the probed atom, since the kinetic energy of the detected electron is effected by such potential^20^,^21^,^22^,^23^,^24^,^25^,^26^,^27^,^28^,^29^,^30^,^31^,^32^. AES was used to probe the electrical potential variations across a working device almost three decades ago, but was not pursued due to its limited chemical specificity^33^,^34^,^35^. Successful reports on the study of the potential distribution across a p-n junction device using PEEM (photoemission electron microscopy) have also been published; similar to the AES also the chemical specificity of PEEM is limited^36^,^37^,^38^. A complimentary approach of the PEEM technique is the scanning photoelectron microscopy/spectroscopy (SPEM/S), which requires the limitedly available and highly coherent synchrotron based x-ray radiation. It is a promising technique providing both superb energy and spatial resolution, down to approximately 0.1 eV and 0.1 μm, respectively. While SPEM/S investigation across a Si p-n junction revealed Si2p core-level shifts on the order of <1 eV as a result of both dopant related and band-bending effects^28^,^39^,^40^, a remarkable 2.1 eV difference was reported in the Ga3d peaks of a GaN p-n junction^41^,^42^. However, an extension to the characterization of relevant devices under operational conditions has not been fully realized yet.

Applications toward investigating devices under more realistic operational conditions are now being reported as a consequence of the recent advances in ambient-pressure XPS (APXPS)^43^,^44^,^45^,^46^,^47^. In parallel, recent advances in commercial XPS instrumentation accessibility and capabilities, such as a micro-focusing and parallel detection systems, have also provided new possibilities to acquire XPS data with a higher lateral resolution and good statistics in reasonable measurement times^48^. Use of XPS for investigating materials and especially device performance under operational conditions, the so-named in-operando XPS is attractive, since the measurement of the binding energy shifts provides information related to the local electrical potential of the chemically identified species.

We previously presented an XPS investigation of a CdS-based photoresistor under working conditions^49^, where the electrical potential variations across the device were mapped by recording binding energy positions under an applied +6 V DC bias across the electrodes, and with and without laser illumination at different wavelengths. Cd3d_5/2_ peak positions were used to calculate the electrical parameters and detect the presence of morphological defects. Another study focused on a CVD grown resistive graphene layer between two gold electrodes with a current flow through the device. In this case the procedure allowed us to detect the presence of defects and cracks when the graphene layer was subjected to a mild oxidation^50^. Furthering that study, we also reported on an investigation where gate-tunable photoemission of a graphene transistor was presented, for the graphene layer as well as the Si_3_N_4_ substrate^51^. We also reported on an investigation of a Si p-n junction during its operation under forward and reverse biases^52^ and, most recently, on the chemical visualization of a GaN p-n junction on the entire surface (2 dimensional) of the device during its operation without and under illumination. The present work extends such investigations to a more complex transistor-like architecture of a bipolar p-n-p junction. The results will demonstrate the power of chemical imaging by XPS for locating and shifting the position of the junction with respect to the polarity of biasing, while the device is in different operation modes. Moreover, we will also demonstrate that, the effect of back-contacting of the device and exposure to harsh chemical treatments, can also be followed in a chemically addressed and also laterally resolved fashion. To our knowledge, this is the first time that such an investigation is presented. Details of the electrical performance of the device, and use of XPS under different biasing configurations, will be given in the Supporting Information (SI) section.

## Results

**Figure 1:**
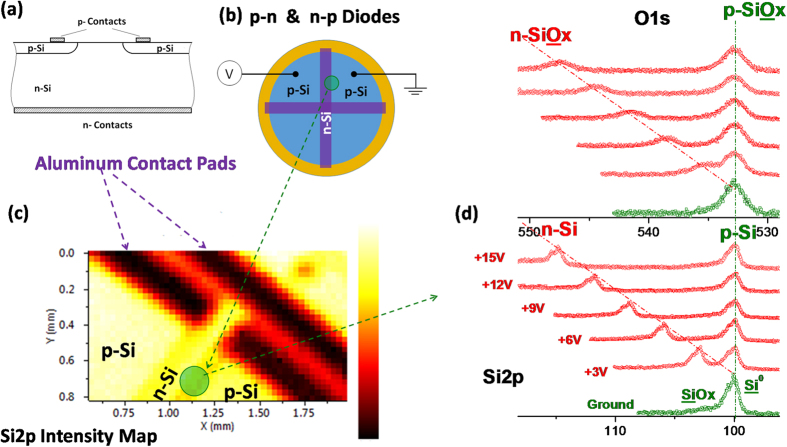
Schematics of the device and its electrical performance under DC bias. (**a**) Schematics of the device and (**b**) its electrical contacts from a cross-section and a top view, respectively. (**c**) A regional areal intensity map of the Si2p peak recorded using the snap-shot mode with 30 μm lateral resolution. (**d**) XP spectra of Si2p and O1s regions recorded with a 400 μm spot covering an intersection area between the right side p- and n-regions, when the device was grounded, and under application of various reverse biases for the n-p junction.

The device used in this work is a p-n-p junction device, and has been fabricated by processing two p-doped segments onto an n-doped Si substrate, followed by aluminum contact deposition which are schematically shown as a cross-section and a top view in Fig. 1(a,b). A regional map of the Si2p peak intensity with 30 μm x-ray spot and 30 μm step sizes recorded in the snap-shot data collection mode is depicted in Fig. 1(c). In our experiments, the second (right-hand side) p-doped segment is always grounded, while we apply a varying bias to the first (left-hand side) p-doped segment and n-doped (middle) segment, thereby establishing forward or reverse bias conditions at the p-n and n-p junctions.

**Figure 2:**
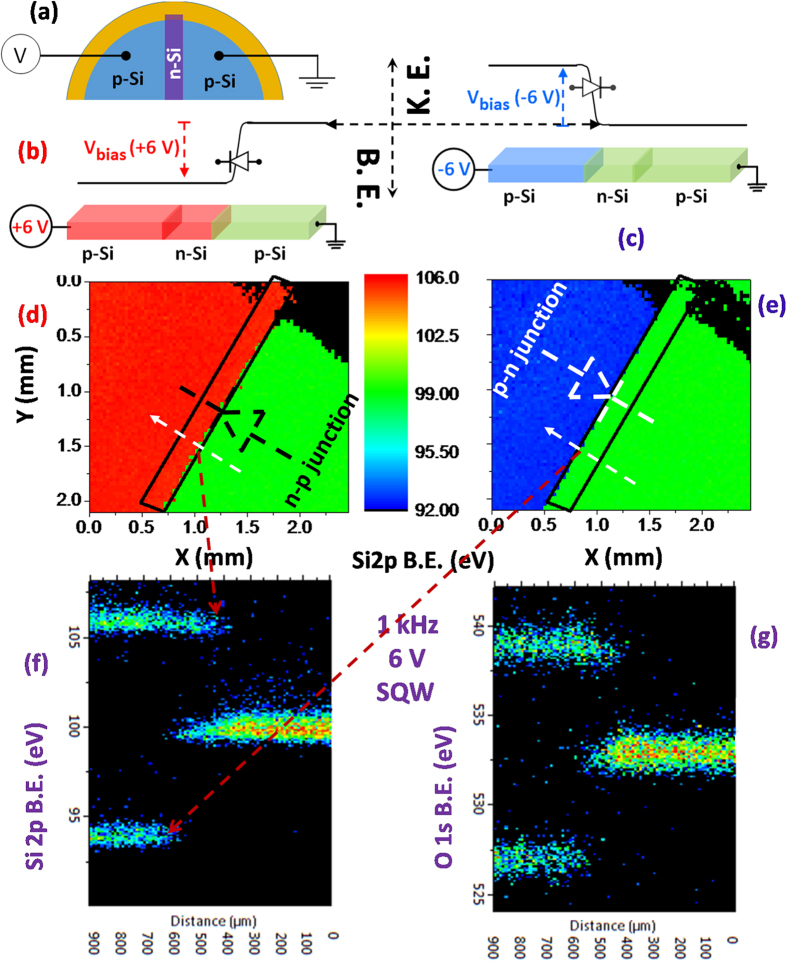
XPS images and spectra indicating the formation of junction and its position shift. (**a**) Schematic representation of electrical connections of the device. Equivalent potential distribution and junction formation of p-n and n-p over three segments of the device under (**b**) +6 V and (**c**) −6 V biases. The color code depicts corresponding potentials on the segments. Areal maps of the measured binding energy position of the Si2p; (**d**) under +6 V, (**e**) under −6 V biases. The black rectangle indicates location of n-segment, and position and direction of diode symbols specify formation of type of junction. The scale bar depicts the color code of the binding energy between 92.0 to 106.0 eV. Note that the location of the junction shifts ~200 μm from one interface to the other when the polarity of the applied bias changes. Spectra of the Si2p and O1s recorded in the line scan mode, along the designated lines shown in (**d**,**e**), under ±6 V amplitude and 1 kHz SQW, are given in **(f**,**g)**, respectively.

First, we apply a positive bias to the first p-doped segment and keep the n-doped segment floating. In this case, the applied bias drops across the reversely biased n-p junction. Note that, although our ultimate lateral resolution of 30 μm does not permit direct investigation of the depletion zone of the junction itself, which is reported to be a few 10 nm^1^,^4^,^5^,^6^,^7^,^8^,^9^,^10^, its location and function can still be probed by XPS, as shown in Fig. 1(d). This figure depicts the Si2p and the O1s peaks, recorded with a regular energy-scanning mode of the instrument, when all segments are grounded and under changing the reverse bias between +3 to +15 V. The 15 V is actually not the upper limit dictated by the device, but higher voltages would significantly alter the collection efficiency of the electron analyzer optics and, therefore, would cause unnecessary spectral artifacts. The x-ray beam is focused in a region that covers both the n- and p-segments (right-hand side), such that when the sample is grounded, both the Si2p and O1s peaks are composed of components from both p- and n-segments. Whereas the difference in the binding energies of the p- and n-doped Si segments, as well as their oxides cannot be resolved when grounded from both sides, both peaks split into two separate components, and the n-component (and as well p-component of the first segment, that is also illustrated in Fig. 2(b,d)) starts to linearly shift in the binding energy as a function of the reverse bias. However, the Si2p and O1s peaks belonging to the right-hand side p-segment stay in the grounded peak position. The surprising finding is that the oxide peak of the native oxide (and possibly also contamination) layers gets broadened and experiences exactly the same energy shift magnitude as that of the Si2p peak. This behavior is representative of the entire n-p junction lines of the device, and the broadening is most probably due to differential charging of the dielectric layer^52^.

### Shift of the Lateral Position of the Junction with Polarity

Unlike a single p-n junction where forward biasing causes current flow, hence no potential development and splitting of the peaks could be observed^53^, the present configuration also allows us to detect the junction formation under both forward and reverse bias configurations as shown in Fig. 2(b–e). Accordingly, although the Si2p position of the grounded right-hand side stays always at 99.5 eV, on the left hand side, the corresponding values become 105.5 and 93.5 eV, under +6 V and −6 V biases, respectively. Overall, a total difference of 12.0 eV is measured complying faithfully with voltage swing, with very small fluctuations on the entire left-hand p-segment. Equally importantly, a ca. ~200 μm lateral shift of the position of the voltage drop, from the p-n junction to the n-p junction, accompanies these binding energy position shifts, as shown in Fig. 2(d,e).

### Square-Wave-Excitation

AC biasing can also be applied to tap other properties of devices. As an example, recording the Si2p and the O1s peaks along a line in the snap-shot mode crossing both junctions, p-n and n-p, under ±6 V (12 V peak-to-peak) and 1 kHz square-wave excitation (SQW) are shown in Fig. 2(f,g), respectively. For the present device, the application of 1 kHz SQW is simply equivalent to applying a positive and a negative DC biases simultaneously, but resulting in a spectroscopically clearer demonstration of the lateral shift of the junction from the n-p to the p-n character. Another advantage of such an application is, in addition to saving time, its parallel detection instead of a serial one, allowing to cancel out many unavoidable experimental imperfections. Furthermore, although not applicable to the present device, by varying the frequency of the SQW, the time-dependent response of certain peaks can also be obtained for certain materials and devices, as was demonstrated in our previous publications for devices made out of photosensitive materials like CdS^54^ and GaN^55^.

### Triangular-Wave-Excitation

**Figure 3:**
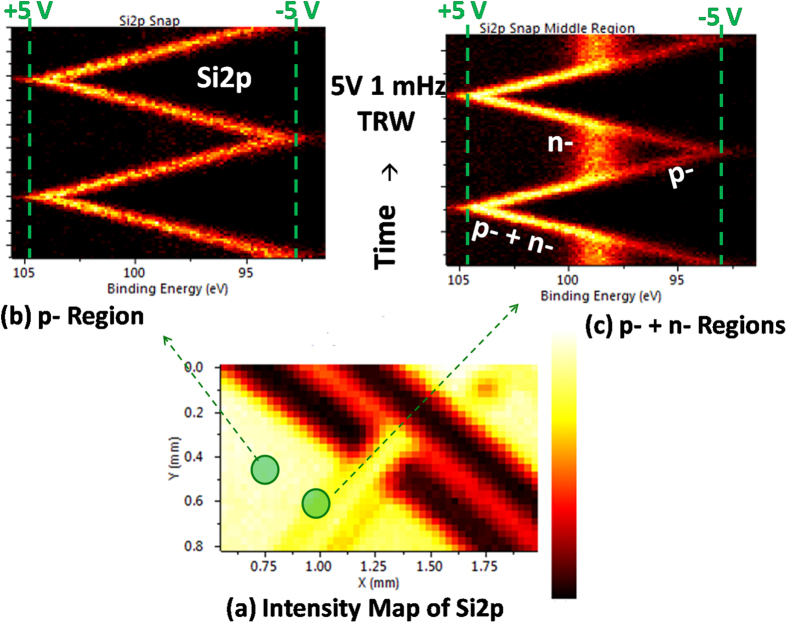
XPS measuerements under TRW excitation from top. (**a**) Si2p areal intensity map. (**b**,**c**) time-varying Si2p spectra recorded under ±5 V and 1 mHz TRWfrom top (left-hand side p-segment) at two different positions, indicatedin (**a**).

Another useful AC bias wave pattern is the triangular wave (TRW) where the voltage is swept slowly between two values, while the relevant peaks are recorded in the snap-shot mode, as was done in our previous work^53^,^56^. The result of it, under normal operational conditions, is a simple and gradual displacement of the peak positions in time, as shown in Fig. 3(b) for the Si2p spectrum recorded at the first (left-hand side) p-segment of the device. When the same data are recorded in the p-n junction region, the situation changes drastically, since the junction is forward-biased during the positive cycle, but reverse-biased starting from near-zero and during the negative cycle. In the latter case, the n-doped segment is at ground potential due to the forward biased n-p junction toward the second (right-hand side), grounded p segment. The Si2p peak representing this region stalls and keeps its position until the negative cycle is completed, whereas the Si2p of the p-region continues to comply with the external bias, as depicted in Fig. 3(c). The two peaks converge again into one in the positive cycle. Since the device has excellent diode properties across the p-n and n-p junctions, no performance degradation via current leaks, etc. is observable, when the n-doped Si segment is floating, as opposed to the case which will be discussed next.

### Three-terminal configuration with TRW Excitation

**Figure 4:**
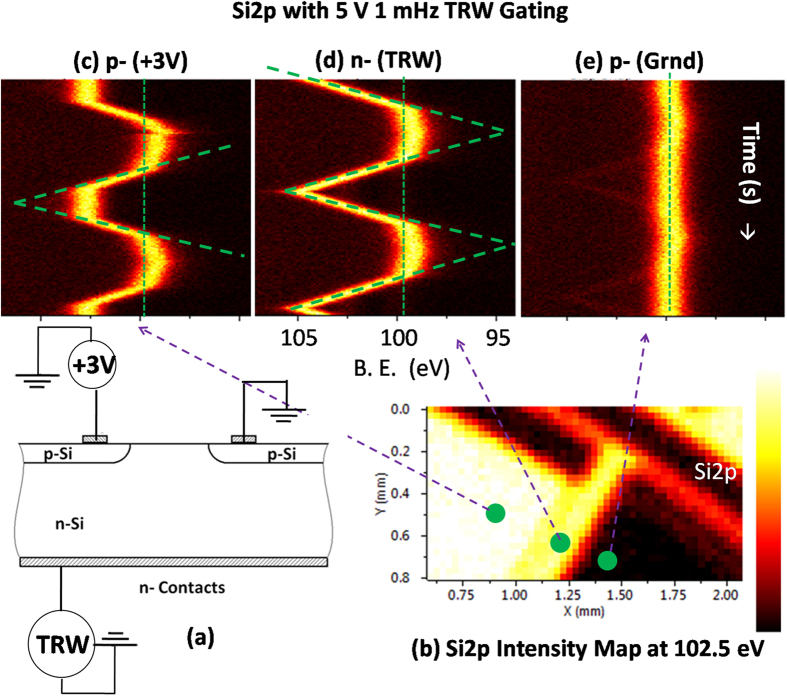
XPS measurements wit back-contacting the device with TRW from the bottomand +3 V DC from the top. (**a**) Shows the electrical connections. (**b**) Si2p areal intensity maps under +3 V bias measured at102.5 eV specific binding energy. Time-varying Si2p spectra, recorded under ±5 V amplitude and 1 mHz TRW and +3 V back-contacting, at three different positions indicated, are shown in (**c**–**e**).

The TRW excitation can also be utilized to follow the current path(s) through the back contacting of the device at the n-doped segment as schematically shown in Fig. 4(a), while imposing a +3 V DC bias through a small button battery to one of the p-segments. In this case, a triangular wave of ±5 V amplitude (10 V peak-to-peak) with 1 mHz frequency was chosen for spectral clarity, and Si2p data were gathered at three representative lateral positions as shown in Fig. 4(c–e). The point (e) corresponds to the grounded right-hand side p-segment of the device and, therefore, almost no variations can be observed, whereas variations in the other two points are quite different. When the back-contact is at +5 V, both diodes are reverse biased and each three segments present the applied potentials, such that no major current is drawn from the circuit except for the low leakage currents flowing through numerous secondary paths. As the TRW voltage gradually falls below +3 V, the p-n diode becomes forward biased and a current starts flowing across the junction. Therefore, the voltage drop is measured on the left-hand p-side, which is connected to the +3 V battery, as a shift in the position of the Si2p peak toward low binding energies, as can be seen in Fig. 4(c). Accordingly, the measured voltage now follows the TRW excitation, most probably due to the capacity fading of the small button battery, and almost diminishes near zero potential. This situation is not applicable to the middle n-region, as given in Fig. 4(d), which is not influenced by the +3 V applied and its peak position monotonically follows the voltage drop of TRW excitation up to reaching zero potential. This behavior is similar to what was shown for the n-component in Fig. 3(c). Finally, the grounded p-region, depicted in Fig. 4(e), stays at the grounded peak position of Si2p all the time.

### Performance Checks of the Damaged Device

**Figure 5:**
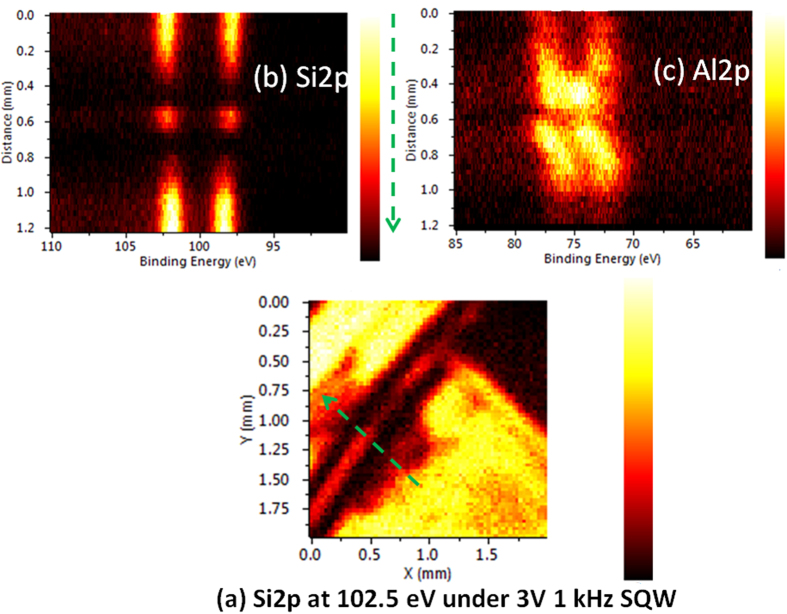
XPS Si2p map and spectra after severely damaging a region. XP spectra and map of Si2p peakunder ±3 V amplitude and 1 kHz SQW excitation from top (left-hand side p-segment). (**a**) Intesity map at the 102.5 eV specific binding energy, and (**b**) Si2p and (**c**) Al2pline spectra of the sample recorded along the line designatedin (**a**).

Another important issue of this study is related with assessing the performance of devices under harsh conditions like in the space environment, or after exposure to hazardous chemicals^57^. Here again, local electrical properties as determined by XPS in a chemically addressed fashion and under operational conditions can be unique, since such information is not derivable by standard electrical measurements. For this purpose we have firstly exposed the analyzed regions of device to 3 kV Ar^+^ etching in vacuum for several minutes, which only resulted in cleaning of the native oxides of both the aluminum electrodes and the silicon, and brought out no new spectral change. Therefore, our next treatment consisted of exposure of a certain spot to a concentrated H_2_O_2_ solution for 60 s, after which the device was washed, dried, and inserted into the transfer chamber of the instrument in atmospheric ambient. Figure 5(a) shows the Si2p aerial map, where the severely damaged regions are clearly visible. Surprisingly the overall function of the device was not affected by this harsh chemical treatment, evident from the well-separated components of the Si2p under a ±3 V amplitude and 1 kHz SQW excitation. The line scan spectra of the Si2p also confirm the undamaged operation of the device as shown Fig. 5(b,c). The only detrimental effect can be seen in the Al2p spectra of the partially damaged contact pad shown in the same figure.

In summary, we have presented the use of a chemical analysis tool, XPS, to extract some of the electrical properties of a device having both p-n and n-p junctions, by applying different biasing configurations to mimic its realistic operational conditions. Switching the polarity of the DC bias causes a shift in the lateral position of the voltage drop across the p-n-p device, which is visualized by the shift in the binding energy of the Si2p. This lateral position shift could also be realized by use of a 1 kHz SQW excitation. Use of TRW excitation of either the top p-segment and the back-contacted n-segment of the device allowed us to trace and analyze the current paths at laterally different positions. Finally, the assessment of the effects of a harsh chemical treatment was also possible at different locations in a chemically addressed fashion. All of these findings are unique and can be pivotal for the characterization of both materials and devices with respect to the otherwise impossible performance- and/or failure-analyses. To this end we would like to emphasize that the main trust of the present work was only the demonstration of these novel applications of XPS, as a proof-of-principle. Our future work will focus on investigations of charge accumulation and dissipation, and probing photo-excitation effects under flat-band and/or band-bending conditions at or near the junction(s), using both lab-based XPS, as well as synchrotron-based facilities with better lateral and spectral resolution.

## Methods

### Device Fabrication

The fabrication of the device is performed using a 500 μm thick n-type doped Si wafer having a resistance of >10 kOhm. The wafer is wet oxidized to obtain a 1 μm thick masking layer for diffusions. First, the backside SiO_2_ layer is removed by wet etching. Then phosphorus diffusion is performed by solid sources at 1100 °C for 75 minutes resulting in a diffusion depth of 4 μm with a 10^21^ atoms/cc surface concentration. After capping back side with plasma-enhanced chemical vapor deposition (PECVD) grown Si_3_N_4_, the front side processing starts. After defining the diffusion areas for p-type dopants by standard photolithography techniques, diffusion windows are removed by dry etching with inductively coupled plasma – reactive ion etching (ICP-RIE) technique. P-type diffusion is performed also at 1100 °C with solid diffusion sources for 75 minutes which results in a approximately 3.5 μm deep diffusion with a 10^20^ atoms/cc surface concentration. Removal of the diffusion mask and back side capping layer is done by wet etching. Aluminum contacts are evaporated to the back side and photolithographically defined areas on the front side of the wafer by e-beam evaporation. Following the lift off, the sample is cleaved and prepared for XPS analysis.

### XPS Measurements

A Thermo Fisher K-Alpha electron spectrometer with monochromatic AlKα X-rays (1486.6 eV) was used for the XPS analyses; standard sample holder was modified to allow the application of one or two external voltage biases across the sample during data acquisition. Two different acquisition modes are used for gathering data; (i) the slower traditional energy scanning mode with better energy resolution, and (ii) the faster snap-shot mode with poorer one. The instrument also provides focused X-ray beam spot with varying sizes from 30 to 400 μm. Depending on the information sought, data is gathered at a single point, or along a designated line, or in an aerial mapping fashion. The Avantage software program of the instrument is used to fit and analyze the data, which are presented in forms of either spectral display of certain peaks or computed parameters, i.e. energy position and intensity of peaks. External voltage bias is also imposed in several modes, such as; (i) positive or negative DC, (ii) square-wave (SQW) excitation with varying amplitude and frequency, and (ii) triangular-wave (TRG) excitation with varying amplitude and frequency. Typical examples of data acquired under these excitations are presented in the Supporting Information (SI) section. For imposing a second bias for implementing the back-contacting configuration, a sealed 3 V button-battery is incorporated to the sample holder, and is also inserted into the vacuum chamber together with the device.

## Additional Information

**How to cite this article**: Copuroglu, M. *et al*. Location and Visualization of Working p-n and/or n-p Junctions by XPS. *Sci. Rep*. **6**, 32482; doi: 10.1038/srep32482 (2016).

## Supplementary Material

Supplementary Information

## References

[b1] Sze S. M. & Ng K. K. Physics of Semiconductor Devices 3rd edn (John Wiley & Sons, 2007).

[b2] Ohl R. Silicon P-N Junction. PBS Online. (https://en.wikipedia.org/wiki/Russell_Ohl) *Retrieved October* 19 (2015).

[b3] Chang T. H. P. & Nixon W. C. Electron beam induced potential contrast on unbiased planar transistors. Solid State Electron. 10, 701–704 (1967).

[b4] Kaestner B., Schönjahn C. & Humphreys C. J. Mapping the potential within a nanoscale undoped GaAs region using a scanning electron microscope. Appl. Phys. Lett. 84, 2109–2111 (2004).

[b5] Heath J. T., Jiang C.-S. & Al-Jassim M. M. Measurement of semiconductor surface potential using the scanning electron microscope. J. Appl. Phys. 111, 046103 (2012).

[b6] Li J. . Direct Identification of Metallic and Semiconducting Single-Walled Carbon Nanotubes in Scanning Electron Microscopy. Nano Lett. 12, 4095–4101 (2012). 22730928 10.1021/nl301561f

[b7] Shibata, . Imaging of built-in electric field at a p-n junction bu scanning transmission electron microscopy. Scientific Reports 5, 10040 (2015). 26067359 10.1038/srep10040 PMC4464396

[b8] Lu Y.-J. . Dynamic Visualization of Axial p–n Junctions in Single Gallium Nitride Nanorods under Electrical Bias. ACS Nano 7, 7640–7647 (2013). 23915124 10.1021/nn4034986

[b9] Tchoulfian P. . Direct Imaging of p–n Junction in Core–Shell GaN Wires. Nano Lett. 14, 3491–3498 (2014). 24837761 10.1021/nl5010493

[b10] Zhou X., Lu M.-Y., Lu Y.-J., Gwo S. & Gradečak S. Correlation of doping, structure, and carrier dynamics in a single GaN nanorod. Appl. Phys. Lett. 102, 253104 (2013).

[b11] Deb P. . GaN Nanorod Schottky and p−n Junction Diodes. Nano Lett. 6, 2893–2898 (2006). 17163726 10.1021/nl062152j

[b12] Bilousov O. V. . Fully Porous GaN p–n Junction Diodes Fabricated by Chemical Vapor Deposition. ACS Appl. Mater. Interfaces 6, 17954–17964 (2014). 25271924 10.1021/am504786b

[b13] Buscema M., Groenendijk D. J., Steele G. A., van der Zant H. S. J. & Castellanos-Gomez A. Photovoltaic effect in few-layer black phosphorus PN junctions defined by local electrostatic gating. Nat. Commun. 5, 4651 (2014). 25164986 10.1038/ncomms5651

[b14] Choi J.-H. . Complete gate control of supercurrent in graphene p–n junctions. Nat. Commun. 4, 2525 (2013). 24056682 10.1038/ncomms3525

[b15] Gabor N. M., Zhong Z., Bosnick K., Park J. & McEuen P. L. Extremely Efficient Multiple Electron-Hole Pair Generation in Carbon Nanotube Photodiodes. Science 325, 1367–1371 (2009). 19745146 10.1126/science.1176112

[b16] Pospischil A., Furchi M. M. & Mueller T. Solar-energy conversion and light emission in an atomic monolayer p-n diode. Nat. Nanotechnol. 9, 257–261 (2014). 24608229 10.1038/nnano.2014.14

[b17] Yan K. . Modulation-doped growth of mosaic graphene with single-crystalline p–n junctions for efficient photocurrent generation. Nat. Commun. 3, 1280 (2012). 23232410 10.1038/ncomms2286 PMC3535365

[b18] Zhang Y. J., Ye J. T., Yomogida Y., Takenobu T. & Iwasa Y. Formation of a Stable p–n Junction in a Liquid-Gated MoS2 Ambipolar Transistor. Nano Lett. 13, 3023–3028 (2013). 23795701 10.1021/nl400902v

[b19] Baugher B. W. H., Churchill H. O. H., Yang Y. & Jarillo-Herrero P. Optoelectronic devices based on electrically tunable p-n diodes in a monolayer dichalcogenide. Nat. Nanotechnol. 9, 262–267 (2014). 24608231 10.1038/nnano.2014.25

[b20] Bozzini B. . Microscale Evolution of Surface Chemistry and Morphology of the Key Components in Operating Hydrocarbon-Fuelled SOFCs. J. Phys. Chem. C 116, 23188–23193 (2012).

[b21] Buller R. . (Pb1–xCdx)S Nanoparticles Embedded in a Conjugated Organic Matrix, as Studied by Photoluminescence and Light-Induced X-ray Photoelectron Spectroscopy. Adv. Funct. Mater. 12, 713–718 (2002).

[b22] Cohen H., Maoz R. & Sagiv J. Transient Charge Accumulation in a Capacitive Self-Assembled Monolayer. Nano Lett. 6, 2462–2466 (2006). 17090074 10.1021/nl061749w

[b23] Cohen H., Sarkar S. K. & Hodes G. Chemically Resolved Photovoltage Measurements in CdSe Nanoparticle Films†. J. Phys. Chem. B 110, 25508–25513 (2006). 17166000 10.1021/jp0648590

[b24] Gregoratti L., Mentes T. O., Locatelli A. & Kiskinova M. Beam-induced effects in soft X-ray photoelectron emission microscopy experiments. J. Electron. Spectrosc. Relat. Phenom. 170, 13–18 (2009).

[b25] Itzhaik Y., Hodes G. & Cohen H. Band Alignment and Internal Field Mapping in Solar Cells. J. Phys. Chem. Lett. 2, 2872–2876 (2011).

[b26] Kolmakov A. . Spectromicroscopy for Addressing the Surface and Electron Transport Properties of Individual 1-D Nanostructures and Their Networks. ACS Nano 2, 1993–2000 (2008). 19206443 10.1021/nn8003313

[b27] Mentovich E., Belgorodsky B., Gozin M., Richter S. & Cohen H. Doped Biomolecules in Miniaturized Electric Junctions. J. Am. Chem. Soc. 134, 8468–8473 (2012). 22537250 10.1021/ja211790u

[b28] Phaneuf R. J. . Imaging the variation in band bending across a silicon pn junction surface using spectromicroscopy. J. Appl. Phys. 88, 863–868 (2000).

[b29] Samokhvalov A. . Charge Transfer between a Gold Substrate and CdS Nanoparticles Assembled in Hybrid Organic−Inorganic Films. J. Phys. Chem. B 107, 4245–4252 (2003).

[b30] Thißen A. . Experimental routes to *in situ* characterization of the electronic structure and chemical composition of cathode materials for lithium ion batteries during lithium intercalation and deintercalation using photoelectron spectroscopy and related techniques. Ionics 15, 393–403 (2009).

[b31] Pantel R. Auger voltage contrast depth profiling of shallow p‐n junctions. Appl. Phys. Lett. 43, 650–652 (1983).

[b32] Patterson J. M. & Smith M. C. A Non Contact Voltage Measurement Technique using Auger Spectroscopy. *Reliability Phys. Symp*., *1983. 21st Annual*, Phoenix. New York: IEEE 150–152 (1983).

[b33] Comizzoli R. B. & Opila R. L. Electrical conduction mechanism in semi‐insulating polycrystalline silicon films. J. Appl. Phys. 61, 261–270 (1987).

[b34] Opila R., Marchut L. & Hollenhorst J. Measurement of the surface electrical potential in a planar avalanche photodiode near breakdown. J. Electrochem. Soc. 137, 703–705 (1990).

[b35] Opila R. L. Electron spectroscopies for simultaneous chemical and electrical analysis. Appl. Surf. Sci. 256, 1313–1315 (2009).

[b36] Giesen M., Phaneuf R. J., Williams E. D., Einstein T. L. & Ibach H. Characterization of p-n junctions and surface-states on silicon devices by photoemission electron microscopy. Appl. Phys. A 64, 423–430 (1997).

[b37] Weinelt M. . Electronic structure and electron dynamics at Si(100). Appl. Phys. A 80, 995–1003 (2005).

[b38] Frank L. . The origin of contrast in the imaging of doped areas in silicon by slow electrons. J. Appl. Phys. 100, 093712 (2006).

[b39] Barrett N., Zagonel L. F., Renault O. & Bailly A. Spatially resolved, energy-filtered imaging of core level and valence band photoemission of highly p and n doped silicon patterns. J. Phys.: Condens. Matter 21, 314015 (2009). 21828576 10.1088/0953-8984/21/31/314015

[b40] Lavayssière M., Escher M., Renault O., Mariolle D. & Barrett N. Electrical and physical topography in energy-filtered photoelectron emission microscopy of two-dimensional silicon pn junctions. J. Electron. Spectrosc. Relat. Phenom. 186, 30–38 (2013).

[b41] Kuo C.-T., Lee H.-M., Shiu H.-W., Chen C.-H. & Gwo S. Direct imaging of GaN p-n junction by cross-sectional scanning photoelectron microscopy and spectroscopy. App. Phys. Lett. 94, 122110 (2009).

[b42] Kuo C.-T. . Natural band alignments of InN/GaN/AlN nanorod heterojunctions. App. Phys. Lett. 99, 1221001 (2011).

[b43] Bluhm H. Photoelectron spectroscopy of surfaces under humid conditions. J. Electron. Spectrosc. Relat. Phenom. 177, 71–84 (2010).

[b44] El Gabaly F. . Measuring individual overpotentials in an operating solid-oxide electrochemical cell. PCCP 12, 12138–12145 (2010). 20694225 10.1039/c003581e

[b45] Ghosal S. . Electron Spectroscopy of Aqueous Solution Interfaces Reveals Surface Enhancement of Halides. Science 307, 563–566 (2005). 15681380 10.1126/science.1106525

[b46] Zhang C. . Measuring fundamental properties in operating solid oxide electrochemical cells by using *in situ* X-ray photoelectron spectroscopy. Nat. Mater. 9, 944–949 (2010). 20871607 10.1038/nmat2851

[b47] Feng Z. A. . Fast vacancy-mediated oxygen ion incorporation across the ceria-gas electrochemical interface. Nat. Com. 5, 4374 (2014). 10.1038/ncomms5374 25007038

[b48] Pelissier B. . Parallel angle resolved XPS investigations on 12in. wafers for the study of W and WSix oxidation in air. Microelectron. Eng. 85, 1882–1887 (2008).

[b49] Sezen H., Rockett A. A. & Suzer S. XPS Investigation of a CdS-Based Photoresistor under Working Conditions: Operando–XPS. Anal. Chem. 84, 2990–2994 (2012). 22369585 10.1021/ac300220u

[b50] Kocabas C. & Suzer S. Probing Voltage Drop Variations in Graphene with Photoelectron Spectroscopy. Anal. Chem. 85, 4172–4177 (2013). 23496152 10.1021/ac400489e

[b51] Copuroglu M., Aydogan P., Polat E. O., Kocabas C. & Süzer S. Gate-Tunable Photoemission from Graphene Transistors. Nano Lett. 14, 2837–2842 (2014). 24684262 10.1021/nl500842y

[b52] Suzer S., Sezen H., Ertas G. & Dâna A. XPS measurements for probing dynamics of charging. J. Electron Spectrosc. Relat. Phenom. 176, 52–57 (2010).

[b53] Suzer S. XPS investigation of a Si-diode in operation. Anal. Methods 4, 3527–3530 (2012).

[b54] Sezen H. & Suzer S. Dynamical XPS measurements for probing photoinduced voltage changesCdS. Surface Science 604, L59 (2010).

[b55] Sezen H., Ozbay E. & Suzer S. XPS for probing the dynamics of surface voltage andphotovoltage in GaN. Applied Surface Science 323, 25 (2014).

[b56] Caliskan D., Sezen H., Ozbay E. & Suzer S. Chemical Visualization of a GaN p-n junction by XPS. Scientific Reports 5, 10040 (2015). 26359762 10.1038/srep14091 PMC4566124

[b57] Rho H. . Three-Dimensional Porous Copper-Graphene Heterostructureswith Durability and High HeatDissipation Performance. Scientific Reports 5, 12710 (2015). 26234425 10.1038/srep12710 PMC4522598

