# Supplementary material for: Location and Visualization of Working p-n and/or n-p Junctions by XPS
Source: Sci Rep. 2016 Sep 1;6:32482. doi: 10.1038/srep32482 (PMC5007498; doi:10.1038/srep32482)
Supplement: Supplementary Information [file srep32482-s1.pdf]

Supplementary Information

Location and Visualization of Working p-n and/or n-p Junctions by XPS

Mehmet Copuroglu[a], Deniz Caliskan[b], Hikmet Sezen[a], Ekmel Ozbay[b], and Sefik Suzer*[,a]

[a]Department of Chemistry, Bilkent University, 06800 Ankara, Turkey
[b]Nanotechnology Research Center, Department of Electrical and Electronics Engineering and Department of Physics, Bilkent University, 06800, Ankara, Turkey

*Corresponding author (suzer@fen.bilkent.edu.tr)

<u>Examples for the Different Data Gathering Modes of XPS</u>

Below, in Figure S1, we present the XP spectra of the Si2p spectral region representing a spot size of ~400 µm area, recorded under various external bias applications. The reference value of the binding energy of Si2p is 99.5 eV, subject to slight variations, depending on the type and extent of its doping level. The application of an external d. c. bias simply shifts the kinetic energy, hence the measured binding energy. Accordingly, the applications of -5V and +5V shift it to 104.5 eV and 94.5 eV, respectively, as also measured and shown in Figure S1 (b) and (c). For normal conducting samples, the application of a square-wave pulse is equivalent to the application of a positive and a negative bias simultaneously resulting in doubling of the peaks, but the intensity is divided by two, as shown in Figure S1 (d). Triangular-wave excitation can also be used which requires sequential data collection, hence it is best presented as a 3-Dimensional plot, as shown in Figure S1 (e). One dimension is time, another is the binding energy position, and the third is the intensity of the photoelectron peak.

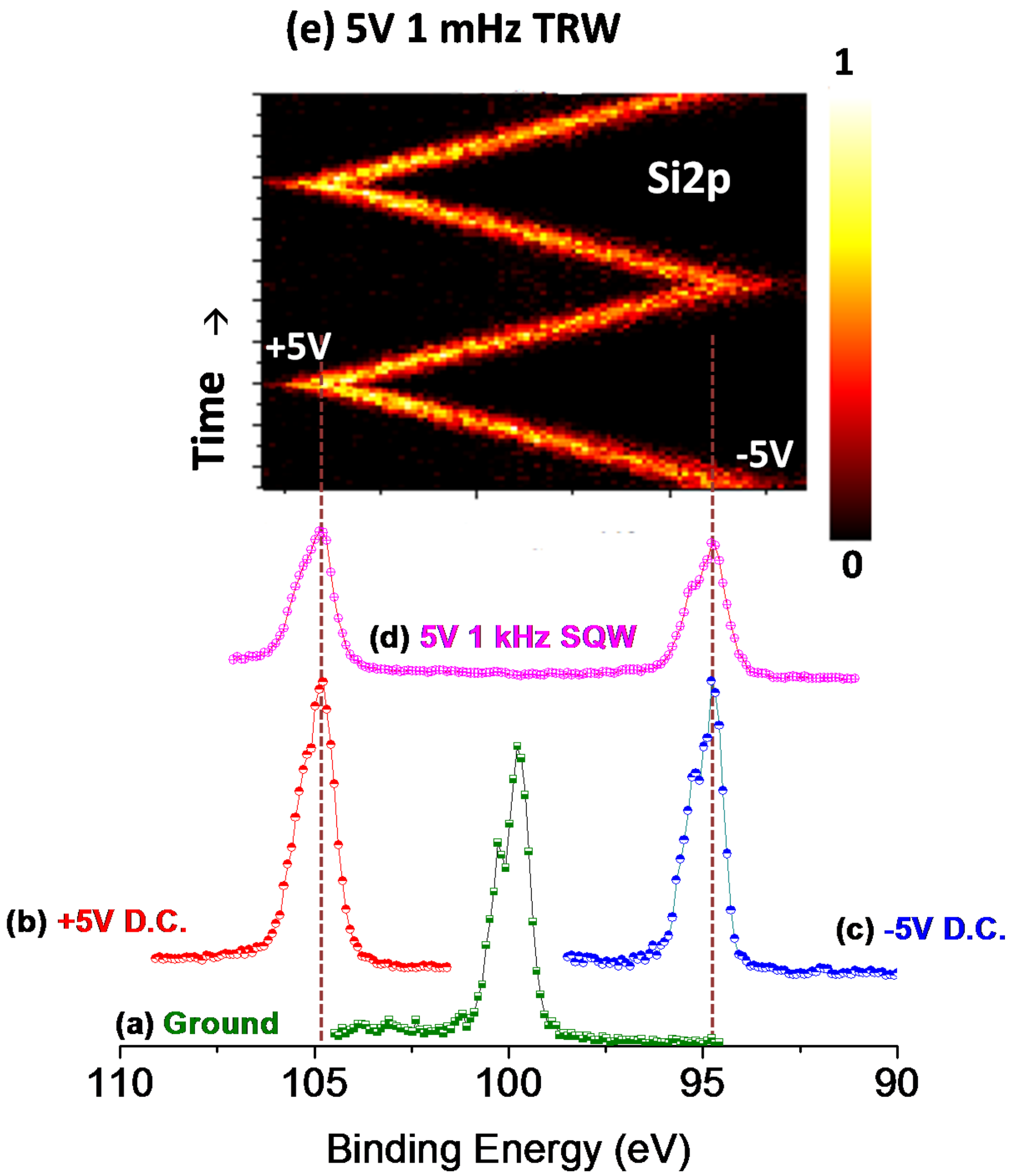

(e) 5V 1 mHz TRW
1
Si2p
Time →
+5V
-5V
0
(d) 5V 1 kHz SQW
(b) +5V D.C.
(c) -5V D.C.
(a) Ground
110
105
100
95
90
Binding Energy (eV)

Figure S1

I-V Data of the Device

The current-voltage characteristics of the device,connected in three different ways and recorded in air-ambient, are shown in Figure S2.

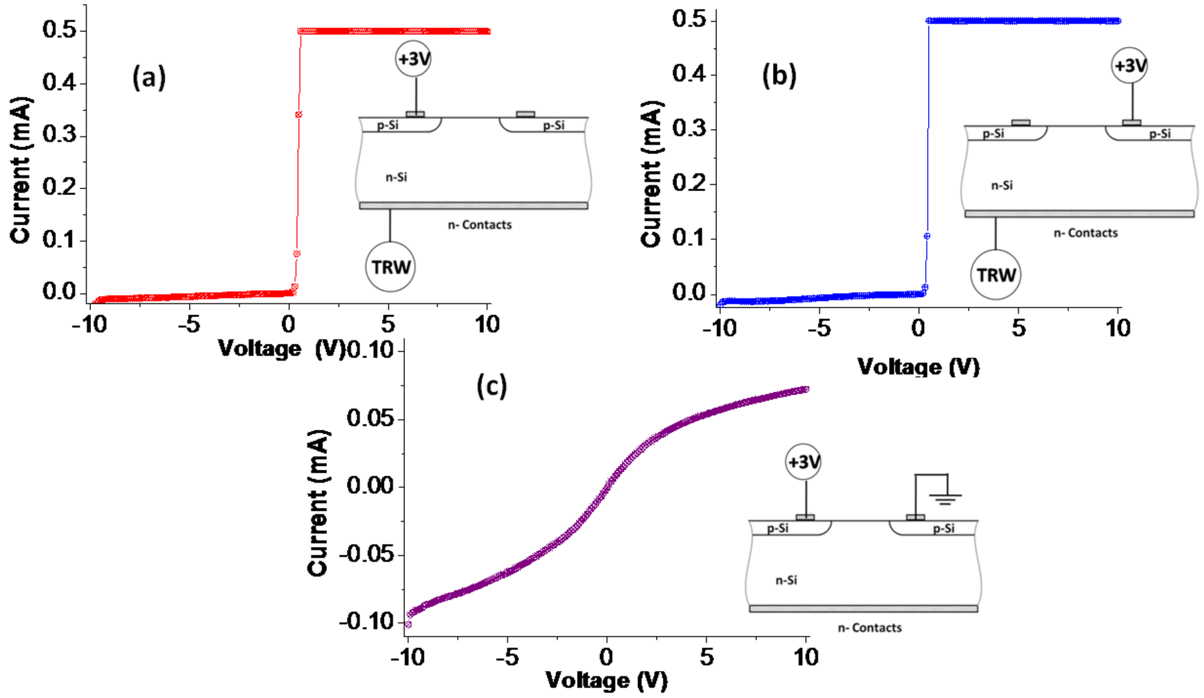

Figure S2
